# Kinetics of free and ligand-bound atacicept in human serum

**DOI:** 10.3389/fimmu.2022.1035556

**Published:** 2022-12-02

**Authors:** Mahya Eslami, Daniela Willen, Orestis Papasouliotis, Sonia Schuepbach-Mallpell, Laure Willen, Olivier Donzé, Özkan Yalkinoglu, Pascal Schneider

**Affiliations:** ^1^ Department of Immunobiology, University of Lausanne, Epalinges, Switzerland; ^2^ Clinical Pharmacology, Translational Medicine, Merck Healthcare KGaA, Darmstadt, Germany; ^3^ Translational Medicine, Merck Institute for Pharmacometrics (an affiliate of Merck KGaA), Lausanne, Switzerland; ^4^ Adipogen Life Sciences, Epalinges, Switzerland

**Keywords:** BAFF, APRIL, heteromers, reporter cells, atacicept

## Abstract

BAFF (B cell activation factor of the TNF family/B lymphocyte stimulator, BLyS) and APRIL (a proliferation-inducing ligand) are targeted by atacicept, a decoy receptor consisting of the extracellular domain of TACI (transmembrane activator and calcium-modulator and cyclophilin (CAML) interactor) fused to the Fc portion of human IgG1. The purpose of the study was to characterize free and ligand-bound atacicept in humans. Total and active atacicept in serum of healthy volunteers receiving a single dose of subcutaneous atacicept or in patients treated weekly for one year were measured by ELISA, Western blot, or cell-based assays. Pharmacokinetics of free and bound atacicept were predicted based on total atacicept ELISA results. Persistence of complexes of purified atacicept bound to recombinant ligands was also monitored in mice. Results show that unbound or active atacicept in human serum exceeded 0.1 µg/ml for one week post administration, or throughout a 1-year treatment with weekly administrations. After a single administration of atacicept, endogenous BAFF bound to atacicept was detected after 8 h then increased about 100-fold within 2 to 4 weeks. Endogenous heteromers of BAFF and APRIL bound to atacicept also accumulated, but atacicept-APRIL complexes were not detected. In mice receiving intravenous injections of purified complexes pre-formed *in vitro*, atacicept-BAFF persisted longer (more than a week) than atacicept-APRIL (less than a day). Thus, only biologically inactive BAFF and BAFF-APRIL heteromers accumulate on atacicept *in vivo*. The measure of active atacicept provides further support for the once-weekly dosing regimen implemented in the clinical development of atacicept.

## Introduction

Atacicept is a fully human, recombinant soluble fusion protein comprising the extracellular ligand-binding domain of the receptor TACI fused to a modified Fc portion of human IgG1 (1), that targets the B cell and plasma cell survival and fitness factors BAFF and APRIL. Atacicept inhibits both soluble and membrane-bound BAFF, soluble APRIL and BAFF - APRIL heteromers (2–4). As a result, atacicept decreases immunoglobulin levels and was associated with higher infection rates compared to placebo, but was not associated with more severe infections, in an analysis of 1586 patients enrolled in double-blind, placebo controlled studies (5). As BAFF and APRIL are implicated in autoimmune diseases (6–11), atacicept has been investigated for its potential to treat these diseases, in particular SLE and IgA nephropathy (1, 6, 12–15). Although the efficacy of atacicept in clinical terms was not as high as hoped for the difficult-to-treat and heterogenous disease SLE (16, 17), high dose atacicept has shown efficacy in two SLE clinical trials: the phase II/III APRILSLE study and the phase IIb ADDRESS II study, both of which showed clinical responses and acceptable safety profiles (13, 15, 18, 19). Atacicept also showed promising results for the treatment of IgA nephropathy (6).

Drug exposure and response can differ between patients. Therefore, studying the exposure-response relationship is important to ensure that drugs administered are safe and effective. In addition, distinguishing between total and ‘free’ drug is important because only the latter can exert a therapeutic effect by binding to and blocking the function of its cognate target(s) (20). Pharmacokinetics of a single dose of atacicept administered subcutaneously in healthy Japanese or Caucasian volunteers was evaluated based on the measure of total atacicept at different time points. Atacicept underwent a rapid absorption phase with maximal serum concentration reached at 20-60 h, a distribution phase of 7 to 10 days and a long terminal elimination phase of longer than 30 days. This study revealed no major ethnicity-based differences (21). In the present study, we have predicted levels of unbound atacicept based on experimental measures of total atacicept, developed an assay to monitor ‘active’ atacicept as a surrogate for unbound atacicept, developed an assay to qualitatively differentiate unbound atacicept in the distribution phase from ligand-bound atacicept in the elimination phase and characterized atacicept-bound endogenous ligands and their effects on the persistence of atacicept in the circulation.

## Materials and methods

### Human samples and study population

Serum samples were obtained from PK samples of the EMR700461-022 (EudraCT ID: 2013-002703-34) and APRIL-SLE (NCT00624338) studies (13). In the phase I EMR700461-022 study, 52 healthy Japanese or Caucasian adults, matched for gender, body weight ( ± 20%), and height ( ± 15%) at screening, were randomized and received atacicept. Subjects were 18–55 years, with a body weight of 45–90 kg (female) and 55–90 kg (male), and a body mass index (BMI) of 18–29.9 kg·m^-2^ (21). In the phase II/III APRIL-SLE study, 455 patients with moderate-to-severe SLE were randomized and received atacicept twice a week for 4 weeks, then weekly for 48 more weeks. Patients were ≥16 years and satisfied ≥4/11 American College of Rheumatology classification criteria for SLE. Patient characteristics have been described previously in more detail (13, 21). Both trials were conducted in accordance with their protocols, the International Conference on Harmonization (ICH) guideline for Good Clinical Practice (GCP) and applicable local regulations, as well as with the Declaration of Helsinki. All study participants provided written informed consent.

### Population PK model

‘Total’ atacicept concentrations of PK samples were used to build a PK model for atacicept, as described (22) (and manuscript in preparation). Briefly, PK data were collected from double-blind, randomized, placebo-controlled clinical studies in which atacicept (25, 75 and 150 mg) was given subcutaneously. These studies were EMR700461-022, APRIL-SLE, and ADDRESS II (NCT02070978) (15, 18). The PK model was a semi-mechanistic nonlinear two-compartment quasi steady-state (QSS) target-mediated drug disposition (TMDD) binding model. ‘Total’ atacicept serum concentrations were determined using a validated ELISA with an acid dissociation step. Non-linear mixed-effect modelling was used to analyze the PK data. Covariates were examined using a stepwise forward selection and backward elimination approach. Final model adequacy was tested by goodness-of-fit and visual predictive check. ‘Free’ atacicept concentration estimates were also obtained with this model (**Supplementary Figure 1**). The software used for the simulation of the profiles included in the manuscript was Berkeley Madonna v 8.3.18 (23). The software used for developing and estimating the population PK model was NONMEM (version 7.3.0) (24).

### Cells and reagents

293T cells were cultured in DMEM, 10% fetal calf serum. Jurkat JOM2-BAFFR : Fas-2308 cl21 and Jurkat BCMA : Fas-2309 cl13 cells have been reported previously and were cultured in RPMI supplemented with 10% fetal calf serum (25–28). Flag-hAPRIL, Flag-hBAFF, Fc-APRIL, Fc-hBAFF, single chain hAPRIL, hBAFF or their heteromers Fc-hAAA, Fc-hBAA, Fc-hABB, Fc-hBBB (produced by transient transfection in 293T cells) were purified on anti-Flag agarose or protein A Sepharose, as described (29). hAAA, hBAA, hABB and hBBB were obtained from their respective purified Fc-PreScission site fusion proteins by digestion with GST-PreScission protease (100 µl protein at 1 mg/ml with 2 µl GST-PreScission at 2 mg/ml (= 2000 U/ml) in PBS for 48 h at 4°C). Plasmids used in this study are described in **Table 1**. Anti-mAPRIL antibody clone Heaty-1 (AG-20B-0082-C100), anti-hBAFF antibodies clone 2.81 (AG-20B-0018-C100), clone 4.62 (AG-20B-0017B-C100), clone 1-35-1 (AG-20B-0037-C100), anti-NAIP 1/2/5 clone Naipa-1 (AG-20B-0045-C100), anti-CD40 clone FGK45 (AG-20B-0036-C100) were from Adipogen (Epalinges, Switzerland). Atacicept and mouse IgG anti-hTACI antibodies Ata1 (E10832) and Ata2 (E10723) were provided by Merck Healthcare KGaA, Darmstadt, Germany. Rat IgM anti-hBAFF Buffy2 was as described (30, 31). Mouse IgG1 anti-hAPRIL Aprily2 was as described (32). PNGase F was from Biolabs (P0704S).

**Table 1:** Plasmids used in this study.

Plasmid	Designation	Protein encoded	Backbone
ps336	Flag-hBAFF	HA signal-Flag-GPGQVQLQ-hBAFF (137-285)	PCR3
ps1361	Flag-hAPRIL	HA signal-Flag-GPGQVQLQ-hAPRIL (98-233)	PCR3
ps1377	pMSCV-puro	Modified pMSCV-puro (Clonetech) with Hindlll-Bglll-EcoRl-Notl-Xhol-Hpal-Apal sites	ps1377
ps1968	GST-PreSci	GST-PreScission	pGEX T2
ps2308	hBAFFR:Fas	HA signal-LE-hBAFFR (aa2-71)-EFGSVD-hFas (aa 164-355)	ps1377
ps2309	hBCMA:Fas	lg signal-VQCEVKLVPRGS-hBCMA (aa 2-54)-VD-hFas (aa 169-355)	ps1377
ps2825	Fc-hBAFF	HA signal-LD-hIgG1 (245-470)-RSPQPQPKPQPKPEPEGSLQ-hBAFF (137-285)	PCR3
ps2826	Fc-hAPRIL	HA signal-LD-hIgG1 (245-470)- RSPQPQPKPQPKPEPEGSLQ-hAPRIL (98-233)	PCR3
ps3670	Fc-AAA	HA signal-LD-hIgG1 (245-470)-RSPQPQPKPQPKPEPEGS-PreSci-LQ-hAPRIL (95-233)-GGGGS-hAPRIL (95-233)-GGGGS hAPRIL (95-233)	PCR3
ps3671	Fc-BAA	HA signal-LD-hIgG1 (245-470)-RSPQPQPKPQPKPEPEGS-PreSci-LQ-hBAFF (140-285)-GGGGS-hAPRIL (95-233)-GGGGS hAPRIL (95-233)	PCR3
ps3672	Fc-ABB	HA signal-LD-hIgG1 (245-470)-RSPQPQPKPQPKPEPEGS-PreSci-LQ-hAPRIL (95-233)-GGGGS-hBAFF (140-285)-GGGGS-hBAFF (140-285)	PCR3
ps3673	Fc-BBB	HA signal-LD-hIgG1 (245-470)-RSPQPQPKPQPKPEPEGS-PreSci-LQ-hBAFF (140-285)-GGGGS-hBAFF (140-285)-GGGGS hBAFF (140-285)	PCR3

HAsignal: MAIIYLIILLFTAVRG. lg signal: MNFGFSLIFLVLVLKG. Flag: DYKDDDDK. PreScission (PreSci): LEVLFQGP.

### Biotinylation

200 µg of Ata1, Ata2, Aprily2 or Buffy2 in 100 µl of 0.1 M Na-borate pH 8.8 were biotinylated with 20 μg EZ-Link-Sulfo-NHS-LC-biotin (Pierce) for 2 h at room temperature. The reaction was stopped by the addition of 10 μl of 1 M NH_4_Cl, and buffer was then exchanged for PBS in a 30 kDa cut off centrifugal device (Millipore).

### Formation of BAFF-atacicept and APRIL-atacicept complexes

Three μg of Flag-hBAFF or Flag-hAPRIL were mixed with 10 μg of atacicept and incubated for 2 h at 37°C. These complexes, as well as Flag-hBAFF, Flag-hAPRIL and atacicept alone, were analyzed as described under “SDS-PAGE and Coomassie blue staining”.

Two mg of atacicept were mixed with 100 µg of Flag-hBAFF, and 3 mg of atacicept were mixed with 150 µg of Flag-hAPRIL. Complexes were purified away from excess unbound atacicept as described under “Size exclusion chromatography” and purified complexes were injected in mice as described under “Mice treatment”.

To estimate the stability of hBAFF/atacicept or hAPRIL/atacicept complexes, 200 µg of atacicept was mixed with 10 µg of Flag-hBAFF or Flag-hAPRIL, then size-fractionated twice as described under “Size exclusion chromatography”.

### Size exclusion chromatography

Flag-hAPRIL, Flag-hBAFF, atacicept or the mix of atacicept with Flag-hBAFF or Flag-hAPRIL were size-fractionated at 0.7 ml/min on a Superdex S200 Increase HR 10/30 column (GE Healthcare) equilibrated in PBS 10 μg/ml BSA, with online absorbance monitoring at 280 nm and 1 ml fraction collection. Fractions 13 and 14 of atacicept alone or fractions 10 and 11 of atacicept/hBAFF or atacicept/hAPRIL complexes were concentrated using 30 kDa cut off centrifugal concentrators. For the estimation of complex stability, atacicept/Flag-hBAFF and atacicept/Flag-hAPRIL complexes were size-fractionated again in PBS 10 μg/ml BSA.

300 µl of serum from an atacicept-treated SLE patient taken pre-dose, or after 4 weeks of treatment, or after 52 weeks of treatment plus 12 weeks of follow-up post treatment were size-fractionated in PBS with 1 ml fraction collection.

The size exclusion chromatography column was calibrated with 100 μl of a mixture of the following proteins, each at 1.4 mg/ml, except ferritin at 0.14 mg/ml, with sizes of: 669 (thyroglobulin), 440 (ferritin), 158 (aldolase), 13.7 (ribonuclease A), all from GE Healthcare), 67 (bovine serum albumin), 43 (ovalbumin), 29 (carbonic anhydrase), and 6.5 kDa (aprotinin) (all from Sigma-Aldrich).

### Mice treatments

Human TACI binds to mouse APRIL and BAFF (33), atacicept blocks BAFF and APRIL in mice (34) and leads to an accumulation of atacicept-bound mouse BAFF in the circulation (35). For these reasons, mice were a relevant model to monitor the persistence of atacicept/ligand complexes. Mice were handled according to Swiss Federal Veterinary Office guidelines. Experiments with animals performed in this study were approved by the Office Vétérinaire Cantonal du Canton de Vaud (authorization 1370.8 to PS). Female, adult C57BL/6JOlaHsd mice were purchased from Envigo, France. Two to five animals per cage were housed in a specific pathogen-free facility (21°C, 50 ± 10% humidity, 14 h: 10 h light/night cycle), with tunnel kraft, dome, sizzle ball and beech log cage enrichment (Serlab). Mice received water at pH 2.8 and Global Rodent XP18 food (Kliba Nafag). Mice were injected intravenously with 200 µg of atacicept (at 10 mg/kg), or 50 µg of Flag-hBAFF (2.5 mg/kg), or 50 µg of Flag-hAPRIL, or 200 µg of atacicept plus 50 µg of Flag-hBAFF, or 200 µg atacicept plus 50 µg of Flag-hAPRIL. Blood was taken after 15 min, 2 days, and then weekly for 3 to 10 weeks after administration. Mice were also injected with a mixture of pre-formed complexes of Flag-hBAFF/atacicept and Flag-hAPRIL/atacicept that had been purified by size exclusion chromatography. The equivalent of 50 µg of Flag-BAFF and 50 µg of Flag-APRIL, plus bound atacicept, were administrated. In this case, blood was collected after 15 min, 4 h, 1 day, 2 days and one week. Collected blood samples were incubated at 37°C for 2 h and spun at 13000 rpm for 10 min at 4°C. The sera were collected and kept at -20°C until use. Two mice per group were used, because the aim of the experiment was to assess in the same mouse whether persistence of co-administered complexes of Flag-hBAFF/atacicept and Flag-hAPRIL/atacicept would be similar or very different, an experimental setup in which interindividual variability has a very low impact. In this case, randomization and blinding were not used because both mice received the same treatment.

### Chemical coupling of proteins to Sepharose beads

2 mg of Heaty-1, 2.81, Naipa-1 or FGK45 antibodies were coupled to 1 ml of NHS-activated Sepharose beads (GE Healthcare, #90-1004-00). Beads that were stored in isopropanol were centrifuged for 5 min at 2400 *x g* and then washed 3x with 1 ml of ice-cold 1 mM HCl. 1 ml of the antibodies at 2 mg/ml in 0.2 M NaHCO_3_, 0.5 M NaCl, pH 8.3 were added, mixed, and incubated for 30 min at room temperature. Then the beads were washed three times with 1 mL of ethanolamine buffer (0.5 M ethanolamine buffer, 0.5 M NaCl, pH 8.3), then with 1 ml of acetate buffer (0.1 M sodium acetate, 0.5 M NaCl, pH 4) three times and again with 3 times 1 ml of ethanolamine buffer. Beads were incubated for 30 min at room temperature in ethanolamine buffer and again washed with 3 times 1 ml acetate buffer followed by ethanolamine buffer, then acetate buffer and finally PBS. Beads were stored in 1 ml of PBS 0.05% azide at 4°C.

### Immunoprecipitation of BAFF, APRIL and heteromers with anti-hAPRIL mAb Heaty-1 and anti-hBAFF mAb 2.81

10 μl of a 50% slurry of NHS-Sepharose beads coupled to anti-hAPRIL mAb Heaty-1, anti-hBAFF mAb 2.81, or isotype-matched control antibodies NAIP or FGK were added to 50 μl serum of an untreated control, of atacicept-injected subjects or of atacicept-treated SLE patients. Beads were also added to 100 μl of a mix containing atacicept at 5 μg/ml plus Flag-hBAFF, Flag- hAPRIL or single chain hBAFF, hAPRIL or heteromers at 0.5 μg/ml. Beads were incubated overnight at 4°C on a rotating wheel. Samples were centrifuged for 5 min at 2400 x *g*, and depleted supernatants were purified on anti-hAPRIL mAb Heaty-1- or anti-hBAFF mAb 2.81-coupled beads. Beads were washed three times with 100 µl of PBS, eluted with 15 µl of 50 mM Na-citrate pH 2.7 and neutralized with 5 µl of 1M Tris-HCl pH 9. Eluates were analyzed by western blot.

Beads were also added to 100 μl of a mix containing atacicept at 5 μg/ml plus Flag-hBAFF, Flag-hAPRIL or single chain hBAFF, hAPRIL or heteromers at 0.5 μg/ml. The input, eluate and depleted supernatant were kept for western blot or ELISA analysis.

The accumulation of endogenous APRIL on atacicept in sera of treated healthy volunteers was monitored by immunoprecipitating 50 µl of sera at different time points with 10 µl of anti-hAPRIL mAb Heaty-1-coupled beads overnight at 4°C. Beads were washed, and APRIL together with bound atacicept were eluted and neutralized as described above.

### Deglycosylation with peptide N-glycanase F

Endogenous APRIL was purified from 100 µl of pooled sera from healthy volunteers or SLE patients treated with atacicept using 10 µl of anti-hAPRIL mAb Heaty-1-coupled beads. Beads were washed three times with 100 µl of PBS and eluted using 15 µl of 50 mM Na-citrate pH 2.7. The eluate was neutralized with 5 µl of 1 M Tris-HCl pH 9. Half of the eluate (10 µl) or 10 ng of Flag-hAPRIL was deglycosylated using peptide N-glycanase F according to the manufacturer’s recommendations (BioLabs, P0704S). Glycosylated and deglycosylated APRIL were analyzed by western blot under reducing condition and revealed with Aprily2/anti-mouse IgG-HRP and then anti-human-HRP.

### SDS-PAGE and Western blot

SDS-PAGE on 12% acrylamide gels followed by western blot were performed under reducing or non-reducing conditions according to standard procedures and revealed using WesternBright ECL spray (Advansta). Ata1, biotinylated Ata1, biotinylated Ata2, Aprily2, biotinylated Aprily2, Buffy2 and biotinylated Buffy2 were all used at 0.5 μg/ml. Atacicept and non-biotinylated antibodies were revealed with appropriate secondary reagents (horseradish peroxidase-coupled goat anti-mouse IgG, goat anti-rat IgM and goat anti-human IgG at 0.2 μg/ml).

For the analysis of atacicept/BAFF and atacicept/APRIL complexes by Coomassie blue staining, samples were boiled in SDS-PAGE sample buffer for 3 min at 95°C, in the presence or absence of 30 mM of dithiothreitol, then resolved on 12% acrylamide SDS-PAGE and stained with a semidry iD Stain System (Eurogentech).

### ELISA

Human BAFF was measured using Adipogen ELISA kit (AG-45B-0001-KI01), according to manufacturer’s instructions, in the presence or absence of atacicept at 0.001 to 100 μg/ml. BAFF in serum samples was measured with 2 µl serum and in the presence of 3 μg/ml of atacicept (except for **Figures 5C**, **6B** where no additional atacicept was added).

Human APRIL was measured using Adipogen ELISA kit (AG-45B-0012-KI01), according to manufacturer’s instructions, in the presence or absence of atacicept at 1 to 1000 μg/ml. For serum samples, 10 µl were used without addition of atacicept. For sera in fractions of the size-exclusion column, 25 µl were used.

Single chain Fc-hAAA, Fc-hBAA, Fc-hABB (at 10 ng/ml) and Fc-hBBB (at 2 ng/ml), post digestion with PreScission protease, were measured with both BAFF and APRIL ELISAs.

Total atacicept levels were already available. They were measured in human serum using an internal validated ELISA using in-house antibodies and standard Merck Healthcare KGaA, Darmstadt, Germany procedures, as described (21).

### Reporter cell assay

To experimentally measure ‘active’ atacicept, a reporter cell-based assay was developed, based on cytotoxicity assays described previously (26). The JOM2 BAFFR : Fas- 2308 cl21 reporter line, which expresses the hBAFFR : Fas fusion protein, was utilized in these assays (28). Where indicated, the Jurkat BCMA: Fas-2309 cl13 reporter line was also used. Assays were performed in flat-bottomed 96-well plates containing: 10 μl of Fc-hBAFF (or Fc-hAPRIL) at 10-fold the desired final concentration in Roswell Park Memorial Institute medium (RPMI)- 1640 with 10% fetal calf serum (FCS); 40 μl of test serum at a fixed dilution of 1/40 in RPMI 10% FCS; and 50 μl reporter cells at 0.8 to 1.6 x 10^6^ cells/ml. For the standard curve of atacicept, test sera were replaced by 40 μl of atacicept at 2.5-fold the desired final concentration in normal human serum diluted 1/40 in RPMI 10% FCS. Twelve Fc-BAFF standard curves (25–0.008 ng/ml) were added per assay plate, one in the presence of 4 ng/ml atacicept (final concentration), ten in the presence of test samples at a final dilution of 1/100 and the last one with a second concentration of atacicept. Cells were incubated for 16 h at 37°C, 5% CO_2_, after which time cell viability was assessed using the 1-methoxy phenazine methosulfate (PMS)/3-(4,5-dimethylthiazol-2-yl)-2,5-diphenyltetrazolium bromide (MTT) assay and absorbance measurement at 492 nm (26). The EC50 of each Fc-BAFF titration was calculated using the “log(agonist) vs. normalized response – variable slope” function of Prism. EC50 obtained at atacicept concentrations of 0.04, 0.4, 4 and 40 ng/ml were used to produce a standard curve against which active atacicept concentration in test sera was determined.

### Statistical analyses

Normality tests (D’Agostino & Pearson; Anderson-Darling; Shapiro-Wilk; Kolmogorov-Smirnov), concurred to indicate that data at one time point was not normally distributed. Comparison of groups was therefore performed by non-parametric Kruskal-Wallis’ one-way analysis of variance, followed, if positive, by Dunn’s multiple comparison test. Comparison of groups at different time points was performed by two-way analysis of variance followed, if significant, by Sidák’s multiple comparison tests using Prism software (GraphPad Software, version 9). Differences were considered statistically significant when P < 0.05.

### Data availability

Supporting data is available in the Zenodo repository, doi 10.5281/zenodo.7316264.

## Results

### Characterizing the kinetics of ‘active’ atacicept

**Figure 1:**
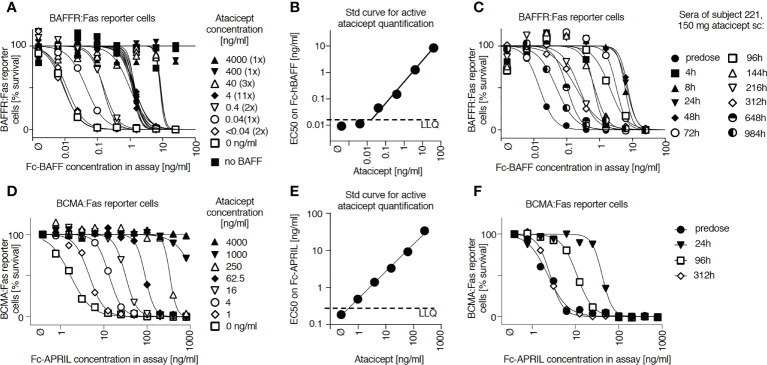
Measure of active atacicept in the serum samples from atacicept-injected subjects with a reporter cell-based assay. **(A)** Increasing concentrations of atacicept can block Fc-BAFF and prevent death of BAFFR : Fas reporter cells. **(B)** Linear relationship between the concentration of Fc-BAFF needed to kill half of BAFFR : Fas reporter cells and the concentration of atacicept. **(C)** Active atacicept concentration in subject’s serum is quantified be measuring the EC50 of Fc-BAFF on BAFFR : Fas reporter cells. **(D–F)** Same as panels **(A–C)**, but using BCMA : Fas reporter cells and Fc-APRIL to measure the ability of active atacicept to inhibit APRIL.

A cell-based assay was devised to monitor ‘active’ atacicept in serum. Fas-deficient Jurkat T cells expressing BAFFR : Fas, a chimeric receptor consisting of the extracellular domain of BAFFR fused to the transmembrane and intracellular domains of the death receptor Fas, underwent a dose-dependent cell death when exposed to recombinant Fc-BAFF. Neutralization of Fc-BAFF by atacicept in human serum protected reporter cells. Thus, higher BAFF concentrations correlated with lower cell survival, while increased atacicept concentrations correlated with increased cell survival (**Figure 1A**). The reporter cell assay was used to generate a standard curve to quantify active atacicept levels in human serum, based on the EC50 of BAFF at different atacicept concentrations (**Figure 1B**). The EC50 of Fc-BAFF correlated linearly with atacicept concentration. This demonstrated validation for the reporter cell assay for the quantification of active atacicept in serum. An example of data obtained from one subject sample (subject 221) at different time points up to treatment day 42 (6 weeks) after a single 150 mg atacicept dose showed that atacicept 150 mg inhibited BAFF and therefore increased cell survival at early time points. Thereafter, protection of reporter cells decreased over time, indicating that the level of active atacicept also decreased over time in subject’s serum (**Figure 1C**). Active atacicept could also be monitored by its capacity to inhibit Fc-APRIL and protect BCMA : Fas reporter cells (**Figures 1D–F**). The sensitivity of this assay was however lower than that using BAFFR : Fas reporter cells, which were used in all subsequent assays.

### Comparison of active vs total atacicept

**Figure 2:**
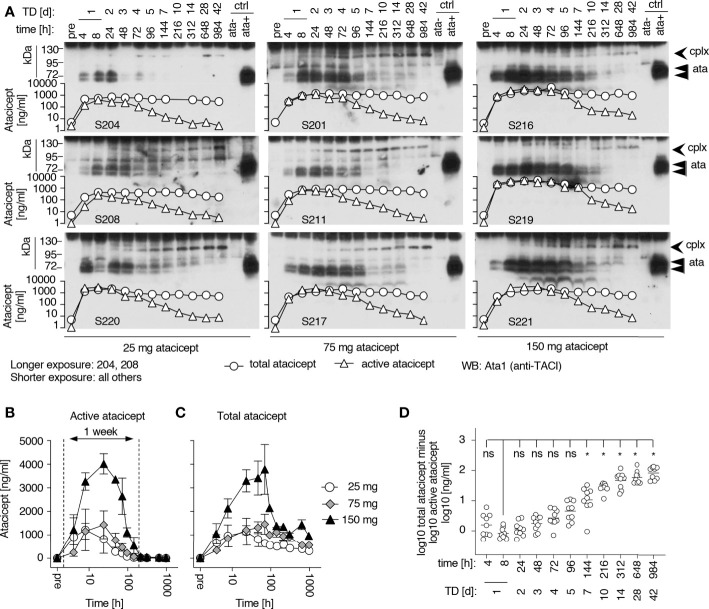
Time versus concentration profiles for subjects receiving increasing doses of atacicept and corresponding Western blot analyses of serum samples. **(A)** Active and total atacicept levels were measured in serum samples of healthy subjects who received a single dose of atacicept (25, 75 or 150 mg). 0.5 µl of serum samples at different time points were also analyzed by denaturing (SDS) but non-reducing Western blot with an anti-TACI (Ata1) antibody. Superimposed graphs show concentrations of active (triangles) or total (circles) atacicept measured in the same samples. cplx: atacicept-containing complex. ata, atacicept. TD, treatment day. **(B)** Active atacicept as a function of time in sera of subjects receiving a single dose of 25 (opened circles), 75 (grey diamonds) or 150 mg (black triangles) atacicept (n=3 per dose. Mean ± SEM). The time interval corresponding to the first week post-administration is shown. **(C)** Same as panel B, but for total atacicept levels measured after acid dissociation of ligands in a validated atacicept ELISA. **(D)** Difference between logarithms of total versus active atacicept concentrations, as a function of time after administration. Non-parametric one-way ANOVA with Kruskal-Wallis’ multiple comparison test. ns, not significant, *p < 0.05. The same set of data was used for all panels. Western blot experiment performed once in this format, and once with TD 0, 4, 14, 28 and 42 for all subjects. Total atacicept measured once. Active atacicept measured twice for all subjects and time points, plus once for TD 0, 4, 14, 28 and 42.

Total atacicept PK profiles determined by ELISA in nine subjects from the EMR700461-022 study were compared with the corresponding active atacicept kinetics determined by the reporter cell assay. Maximal concentrations of active atacicept of ~ 4000 ng/ml were reached at 24 h post single subcutaneous 150 mg dose. Active atacicept then declined and reached levels below 100 ng/ml 144 h post-dose (treatment day 7) (**Figures 2A, B**). Total atacicept also reached a concentration of ~4000 ng/ml at 24 h post 150 mg dose, stayed high until 72 h post-dose, then declined in parallel to active atacicept down to a concentration of ~1200 ng/ml by 144 h post-dose and was eliminated very slowly thereafter to slightly below 1000 ng/ml at the last time point analyzed (*i.e.* 984* h* post-dose, day 42) (**Figures 2A, C**). Similar qualitative observations were made for the 75 and 25 mg atacicept single doses (**Figures 2A–C**).

The time versus concentration profiles of total and active atacicept were compared to atacicept detected by western blot under denaturing but non-reducing conditions (**Figure 2A**). The intensity of the band of atacicept detected in serum by western blotting, which co-migrated with a standard of dimeric ‘free’ atacicept, correlated with active atacicept levels. However, a higher molecular weight band first detected after 1 or 2 days and persisting until after disappearance of dimeric atacicept was hypothesized to represent a signature of ligand-bound atacicept accounting for sustained levels of total atacicept at later time points. This was reproducibly observed in all serum samples. By superimposing the linked profiles, western blot analysis revealed that the absorption and initial distribution phase was characterized by the presence of a majority of ‘free’ atacicept up to the first week post-dose. Thereafter, complexed atacicept gradually dominated in the slow terminal elimination phase (**Figure 2A**). Significant higher levels of total versus active atacicept, measured as difference of log10 concentrations, were found in all 9 healthy volunteers 10, 14, 28 and 42 days post-dose compared to 8 h post-dose, regardless of the dose of atacicept (**Figure 2D**).

**Figure 3:**
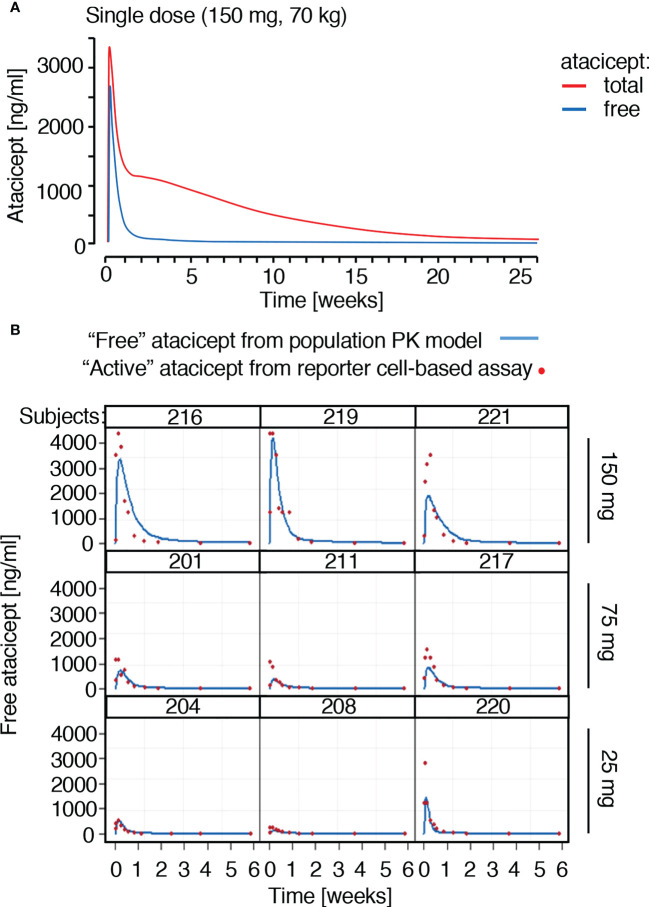
Prediction and measures of free or active atacicept in subjects receiving a single atacicept dose. **(A)** Predicted PK profiles for total and free atacicept for a typical 70 kg subject who received a single dose of 150 mg atacicept. Red line: total atacicept. Blue line: free atacicept. **(B)** “Active” atacicept PK profile (red dots) compared with model-derived “free” atacicept PK profiles (blue line) in nine subjects receiving the indicated doses of atacicept.

**Figure 4:**
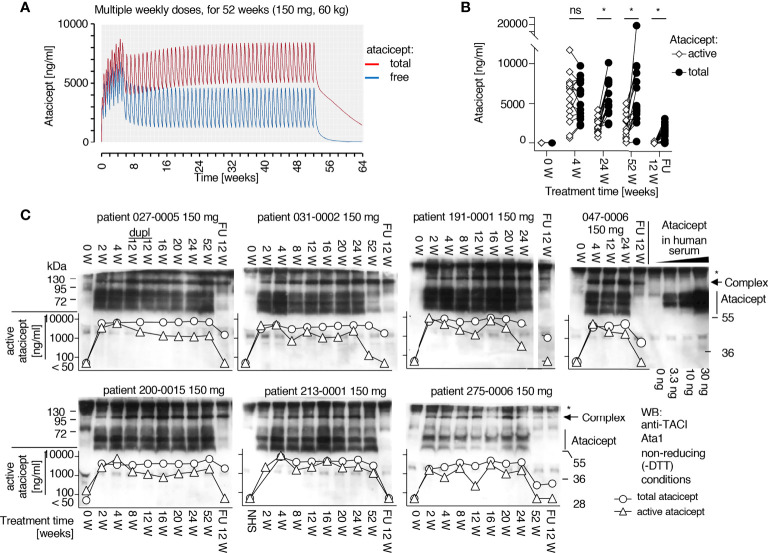
Prediction of free atacicept and measure of active atacicept in SLE patients receiving multiple injections of atacicept. **(A)** Population PK model for a 60 kg subject receiving 150 mg atacicept twice a week for 4 weeks, then weekly for 48 more weeks, with blue and red lines indicating predicted levels of free and total atacicept, respectively. **(B)** Active and total atacicept in 15 SLE patients (10 x 150 mg, 5 x 75 mg), before treatment, at weeks 4, 24 and 52 of treatment, and 12 weeks after treatment end (FU 12 W). Two ways ANOVA with Sidák’s multiple comparison test was used to compare active versus total atacicept at each time point. ns, not significant. *p < 0.05. Experiment performed once. **(C)** Active atacicept measured pre-dose, at the indicated time points during a 1-year atacicept treatment with bi-weekly administration of 150 mg atacicept for 4 weeks, then weekly for 48 further weeks, and at follow-up 12 weeks after treatment end (FU 12 W), in seven SLE patients. Serum samples (1 µl) collected at trough were analyzed in parallel by denaturing, but non-reducing SDS-PAGE followed by Western blot with anti-TACI mAb Ata1. cplx: atacicept-containing complex. ata: atacicept. NHS, normal human serum. Experiment was performed once in this format, and twice with reduced numbers of patients and time points.

A population PK model was developed to best fit total atacicept concentrations in healthy volunteers and patients who had received atacicept (manuscript in preparation). This PK model predicted that free atacicept, passed the maximum concentration after the absorption and initial distribution phase, declines rapidly compared to total atacicept (**Figure 3A**). Free atacicept predicted by the PK model was thus compared with active atacicept levels measured with reporter cells in nine individual subjects from the EMR700461-022 study and found to be in reasonably good agreement across a broad concentration range and the entire PK sampling period (**Figure 3B**). This suggested that reporter cell-determined ‘active’ atacicept can act as a surrogate for ‘free’ atacicept. The PK model for bi-weekly administrations of 150 mg atacicept for 4 weeks, followed by weekly administration for the next 48 weeks predicted free atacicept concentration at trough (*i.e.* just before the next dose) of 3 to 4 µg/ml for the first four weeks, and of about 1.2 µg/ml later in the treatment (**Figure 4A**). Measures of active atacicept at weeks 4, 24 and 52 weeks in 15 SLE patients receiving 75 mg (5 patients) or 150 mg (10 patients) atacicept, and measures of active atacicept at weeks 2, 4, 8, 12, 16, 20, 24 and 52 in seven SLE patients receiving 150 mg atacicept (twice a week for 4 weeks, then weekly for the next 48 weeks) were 5.7 ± 2.8 μg/ml at weeks 2 and 4 of treatment and 2.0 ± 1.3 μg/ml at weeks 8 to 52 of treatment, in reasonable agreement with the model (**Figures 4B, C**). In addition, the SDS-resistant higher molecular band of atacicept hypothesized to represent atacicept complexed with its ligand(s) was detected at all time points. In 6/7 patients, it was also detected at 12 weeks post treatment when dimeric free atacicept had become undetectable (**Figure 1C**). In one patient (275–0006), there was no detectable active atacicept at week 52 of treatment (**Figure 4C**), and low total atacicept (0.25 µg/ml).

### Time-dependent increase of total circulating BAFF after atacicept administration

**Figure 5:**
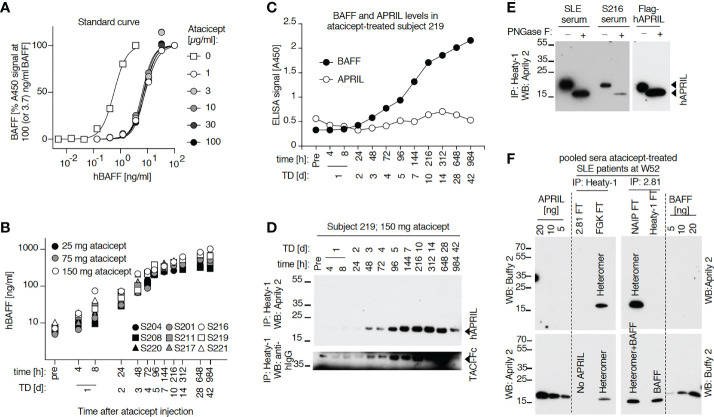
Serum levels of endogenous BAFF and BAFF-APRIL heteromers increase in a time-dependent manner after administration of atacicept. **(A)** hBAFF ELISA signals upon titration of a BAFF standard alone or in the presence of the indicated fixed concentrations of atacicept. This experiment was performed twice under slightly different conditions (including **Figure 6**). **(B)** Healthy subjects received a single dose of atacicept at either 25 (black symbols), 75 (grey symbols) or 150 mg (opened symbols). BAFF levels were quantified in serum samples by ELISA in the presence of an additional saturating level of 3 μg/ml of atacicept. This experiment was performed twice. **(C)** Endogenous BAFF and APRIL levels were measured by ELISA in 10 μl of serum from healthy subject 219 at different time points after atacicept injection, in the absence of additional atacicept in the ELISA assay. (The experiment was repeated once in S219 and S208 at day 0, 1 and 28 after the injection, and also performed for BAFF in S208). **(D)** Endogenous APRIL in 50 μl of serum samples from healthy subject 219, who received 150 mg of atacicept once, was immunoprecipitated with anti-APRIL mAb Heaty-1, then detected by Western blot with anti-hAPRIL mAb Aprily2. TD: treatment day. (The experiment was repeated in S219 at time 312h) (and in S219 using three time points) **(E)** Endogenous APRIL was immunoprecipitated with anti-APRIL mAb Heaty-1 from 100 µl pooled sera samples of subject 219 at treatment days 7, 10, 14, 28 and 42, or from 100 µl pooled sera of five SLE patients at treatment week 52 (4x 150 and 1x 75 mg dose). Half of the elution was left untreated and half was deglycosylated with peptide N-glycanase F (PNGaseF) prior to Western blot analysis with anti-hAPRIL mAb Aprily2. 10 ng of recombinant Flag-hAPRIL was deglycosylated in parallel. The experiment was repeated with pooled samples of subject 208 with similar results. **(F)** Immunoprecipitation of endogenous BAFF and BAFF-APRIL heteromers from pooled sera of SLE patients after 52 weeks of treatment with atacicept. Sera were either pre-depleted with anti-hBAFF mAb 2.81 or isotype control (FGK), then immunoprecipitated with anti-APRIL mAb Heaty-1, or pre-depleted with mAb Heaty-1 (or isotype control NAIP), then immunoprecipitated with mAb 2.81. Immunoprecipitates were analyzed by Western blot with anti-hBAFF mAb Buffy2 or anti-hAPRIL mAb Aprily2, as indicated. Experiment performed once in this format, and once more with IP mAb Heaty-1 followed by IP 2.81.

**Figure 6:**
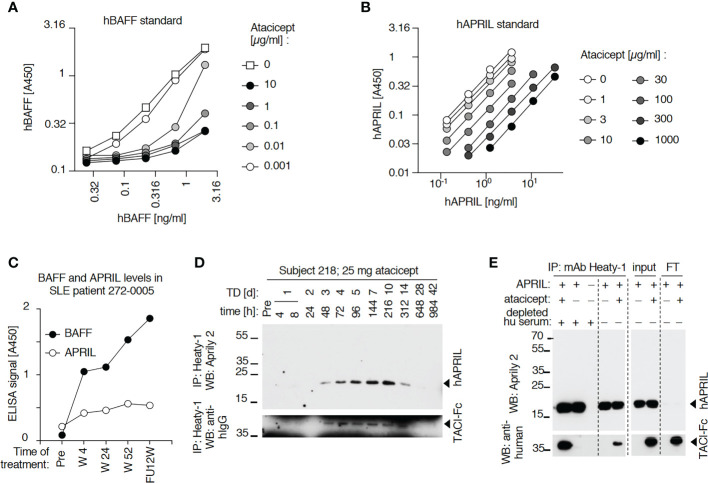
Detection of BAFF and APRIL in sera of an atacicept-treated SLE patient or a healthy subject. **(A)** Detection of titrated amounts of a hBAFF ELISA standard in the presence of increasing concentrations of atacicept. Experiment performed once with these concentrations, and once with higher atacicept concentrations (**Figure 5A**). **(B)** Detection of titrated amounts of hAPRIL ELISA standard in the presence of increasing concentrations of atacicept. Experiment performed twice. **(C)** BAFF and APRIL ELISA signals with 10 µl serum samples of an atacicept-treated SLE patient at different time points. Experiment was performed twice. **(D)** Endogenous APRIL was immunoprecipitated with beads coupled to anti-APRIL mAb Heaty-1 from 50 μl serum samples of healthy subject 208 who received 25 mg of atacicept. Immuno-precipitates were analyzed by SDS-PAGE and Western blot anti-APRIL. Experiment performed once for subject 208, and twice with subject 209 (**Figure 5D**). **(E)** Immunoprecipitation with anti-APRIL mAb Heaty-1 of recombinant APRIL (50 ng), in the presence or absence of atacicept (500 ng), in buffer or in normal human serum pre-depleted from endogenous APRIL. Immuno-precipitates were analyzed by SDS-PAGE and Western blot anti-APRIL to detect APRIL, followed by anti-human IgG to detect atacicept. Experiment performed 3 times.

To monitor endogenous BAFF levels at different time points after a single subcutaneous atacicept injection in healthy volunteers, the serum concentration of this cytokine was measured by ELISA. Although BAFF ELISA signals were reduced in the presence of atacicept, an atacicept-resistant residual signal remained even at high atacicept to BAFF ratio (**Figures 5A**, **6A**). Therefore, endogenous level of BAFF in subjects’ sera were measured in the presence of saturating concentration of atacicept, using a standard curve of BAFF exposed to the same concentration of atacicept (**Figure 5B**). As shown, total endogenous levels of BAFF, which is most likely bound to atacicept, started to rise as early as 4 to 8 h post atacicept administration, then steadily increased about 100-fold within 2 weeks and remained high until the last observation at day 42.

### A form of APRIL that is not recognized by ELISA also accumulates in healthy volunteers or SLE patients that had received a single dose, or multiple doses of atacicept, respectively

APRIL in serum samples was measured by ELISA. Although APRIL signals were partially quenched by atacicept, this interference was modest up to atacicept concentrations of 3 μg/ml and should have allowed qualitative detection of APRIL accumulation in sera after atacicept treatment (**Figure 6B**). Nevertheless, differences in APRIL signal monitored in both a healthy subject and a SLE patient were small compared to those observed for BAFF after atacicept injection (**Figures 5C**, **6C**). However, and in contrast to ELISA data, Western blot analysis did reveal a time-dependent accumulation of atacicept-bound endogenous APRIL protein after atacicept administration in healthy subjects (**Figures 5D**, **6D**). In this experiment, endogenous APRIL was first concentrated by immunoprecipitation with monoclonal antibody mAb Heaty-1 that binds APRIL in buffer or in human serum, regardless of the presence or absence of atacicept and that co-immunoprecipitates atacicept if it is bound to APRIL (**Figure 6E**). The size of endogenous APRIL protein isolated from sera of an atacicept-treated healthy subject or a SLE patient and detected by Western blot was 19 kDa before, and 16 kDa after treatment with peptide N-glycanase F, in close agreement with the presence of one consensus sequence for N-glycosylation and a predicted molecular weight of 16.4 kDa for the naked sequence of soluble mature APRIL (**Figure 5E**). This further confirmed the identity of the immunoprecipitated protein detected by Western blot but raised the question of why APRIL was not detected by ELISA and whether this might be related to the incorporation of APRIL into APRIL-BAFF heteromers.

### Detection of BAFF and APRIL heteromers in SLE patients’ sera after atacicept injection

**Figure 7:**
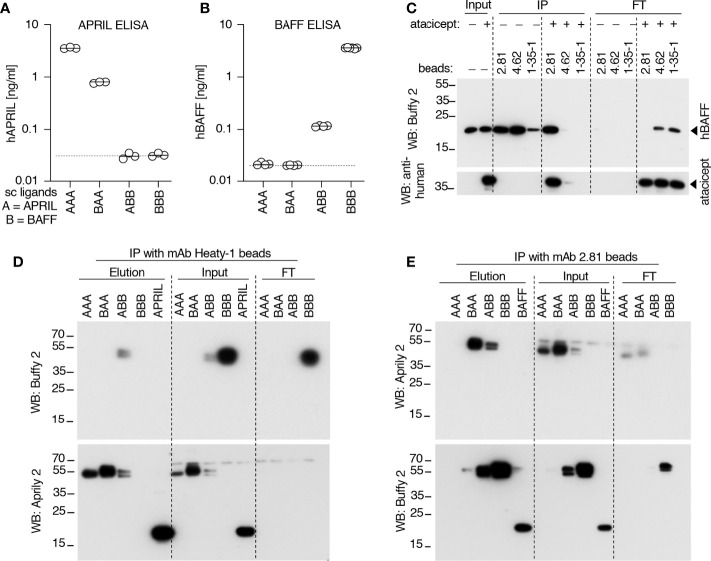
Detection and immunoprecipitation of recombinant BAFF and APRIL heteromers. **(A, B)** The recognition of recombinant Fc-tagged single chain BAFF, APRIL and heteromers was tested in APRIL and BAFF ELISAs. Experiment performed twice. **(C)** Different anti-BAFF mAbs were coupled to beads and used to immuno-precipitate recombinant Flag-BAFF (100 ng) in the presence or absence of atacicept (1000 ng). Immuno-precipitates were analyzed by SDS-PAGE and Western blot anti-BAFF to detect BAFF, followed by anti-human IgG to detect atacicept. Experiment performed three times. **(D)** Single chain recombinant BAFF, APRIL or their heteromers, or Flag-APRIL, were immune-precipitated with beads-coupled anti-APRIL mAb Heaty-1. Immuno-precipitates were analyzed by Western blots anti-APRIL and anti-BAFF. **(E)** Same as panel D, except beads coupled to anti-BAFF mAb 2.81 were used for the immune-precipitation and Flag-BAFF was used as positive control. Experiment was performed twice.

Data so far indicate that after atacicept injection, APRIL protein in serum is detected by Western blot but not by ELISA, suggesting that APRIL might be incorporated into heteromers with BAFF, in line with the observation that heteromers are poorly detected by both BAFF ELISA and APRIL ELISA (**Figures 7A, B**). To investigate this hypothesis, we used beads coupled to anti-hBAFF mAb2.81 that can efficiently immunoprecipitate BAFF, also in the presence of atacicept (**Figure 7C**) and beads coupled to anti-APRIL mAb Heaty-1 that does the same for APRIL (**Figure 6E**) and further characterized their fine binding specificities using molecularly defined single-chain BAFF (BBB), APRIL (AAA), BAFF-rich heteromers (ABB) and APRIL-rich heteromers (BAA) (27). mAb Heaty-1 immunoprecipitated AAA, BAA and ABB, while mAb2.81 immunoprecipitated BBB, BAA and ABB, validating that both antibodies can bind to BAFF-rich and APRIL-rich heteromers (**Figures 7D, E**). Pooled SLE patient sera after 52 weeks of atacicept treatment were sequentially immunoprecipitated first with anti-BAFF mAb2.81 and second with anti-APRIL mAb Heaty-1, or vice versa, and then analyzed by Western blot anti-APRIL and anti-BAFF. mAb2.81 immunoprecipitated BAFF and co-immunoprecipitated APRIL, while mAb Heaty-1 immunoprecipitated APRIL and co-immunoprecipitated BAFF, suggesting that heteromers of BAFF and APRIL were present in these samples. Also, mAb2.81 immunoprecipitated BAFF from serum in which APRIL was pre-depleted with mAb Heaty-1, indicating that part of BAFF in these samples stands alone and is not associated to APRIL. However, mAb Heaty-1 did not immune-precipitate APRIL from serum in which BAFF was first pre-depleted with mAb2.81, indicating that all or almost all APRIL is associated to BAFF and none exists on its own in these sample, or only below the detection limit (**Figure 5F**). These results confirm that BAFF and APRIL are accumulated after atacicept administration, but further indicate that most if not all of APRIL molecules are incorporated into BAFF - APRIL heteromers that cannot be detected by the APRIL ELISA used in these studies.

### Recombinant and endogenous BAFF form SDS-resistant complexes with atacicept

**Figure 8:**
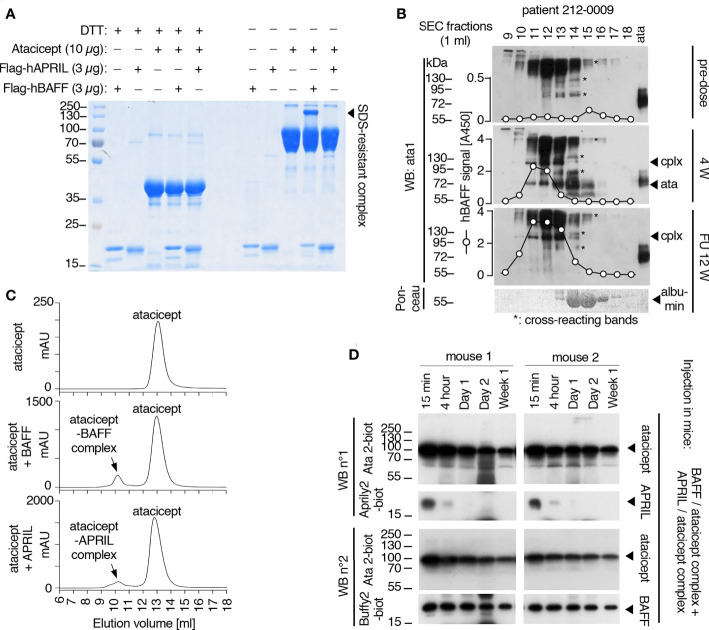
Analysis of BAFF/atacicept complex *in vitro*, in an atacicept-treated human patient serum, and comparison of persistence of BAFF-atacicept and APRIL/atacicept complexes in mice. **(A)** Purified Flag-hBAFF, Flag-hAPRIL, atacicept and complexes thereof were analyzed by SDS-PAGE under reducing (+DTT) or non-reducing (-DTT) conditions, followed by Coomassie blue staining. Experiment performed twice in this format, and four times with BAFF. **(B)** Serum samples from an atacicept-treated SLE patient taken pre-dose, after 4 weeks of treatment (4 W) or 12 weeks after treatment end (FU 12 W) were analyzed by size-exclusion chromatography, followed by Western blot anti-TACI under non reducing conditions. BAFF was also measured in fractions by ELISA. ata: atacicept. cplx: complex ligand-atacicept. “*” indicate bands recognized non-specifically in the pre-dose sample, the heaviest being probably human Ig recognized by the secondary antibody. Experiment performed once. **(C)** Size exclusion chromatography profiles of atacicept alone (300 µg) or of purified Flag-hBAFF or Flag-hAPRIL mixed with a 20-fold mass excess of atacicept to create and isolate atacicept/BAFF and atacicept/APRIL complexes for administration in mice. **(D)** Purified atacicept/BAFF and atacicept/APRIL complexes of panel B were co-administered in two wild type mice. Serum samples collected at the indicated time points were analyzed by Western blot under non-reducing conditions, once for the presence of atacicept and human APRIL and once for the presence of atacicept and human BAFF.

As indicated before (**Figure 2A**), in serum of atacicept-treated individuals, an SDS-resistant complex (of around 100 kDa) accumulated over time that could be detected with anti-TACI mAbs Ata1 or Ata2 under non-reducing conditions. Interestingly, this complex persisted markedly longer than free atacicept. The size suggests that the complex could be composed of an atacicept molecule bound to cognate ligand(s). To test this hypothesis, recombinant Flag-hBAFF or Flag-hAPRIL were pre-incubated with atacicept and formation of the complex was investigated under reducing or non-reducing conditions by SDS-PAGE and Coomassie blue staining. Purified BAFF, but not APRIL, formed the SDS-resistant complex with atacicept under non-reducing condition (**Figure 8A**). In addition, this complex was recognized by both anti-TACI Ata1 and to a weaker extent by anti-BAFF Buffy2 antibodies by western blot (**Supplementary Figure 2A**). In the serum of a SLE patient analyzed by size exclusion chromatography pre-dose, endogenous BAFF monitored by ELISA comigrated with albumin in fraction 15, consistent with the expected size of a soluble BAFF trimer. After 4 weeks of atacicept treatment, BAFF levels increased a lot and shifted to the higher molecular weight fractions 11-13 that also contained the atacicept complex detected by Western blot, while atacicept alone eluted mainly in fraction 14 (**Figure 8B**). This is consistent with the formation of atacicept/BAFF complexes in which the half-live of BAFF is increased by binding to atacicept, but its activity inhibited. Twelve weeks after the end of the 1-year atacicept treatment, serum of this patient still contained a BAFF/atacicept complex in fractions 11-13, while neither free atacicept in fraction 14, nor dimeric atacicept that detaches from the complex upon SDS treatment in fractions 11-13 were detectable, pointing to a remarkable stability of the atacicept/BAFF (or possibly atacicept/heteromers of BAFF and APRIL) complex(es) in the circulation (**Figure 8B**).

### BAFF/atacicept complex has a longer persistence than APRIL/atacicept complex in mice

**Figure 9:**
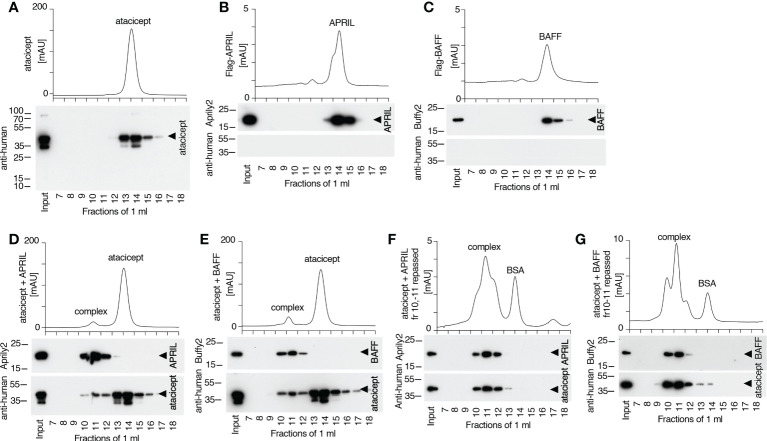
Size and stability of BAFF, APRIL, atacicept and complexes thereof. Size exclusion chromatography profiles of atacicept, Flag-BAFF and Flag-APRIL using PBS 10 μg/ml bovine serum albumin as elution buffer. Fractions were analyzed by Western blot using anti-human IgG antibody (to detect atacicept), anti-APRIL mAb Aprily2 and/or anti-BAFF mAb Buffy2, as needed. **(A)** Atacicept (200 µg). **(B)** Flag-APRIL (10 µg). **(C)** Flag-BAFF (10 µg). **(D)** Atacicept (200 µg) mixed with Flag-APRIL (10 µg). **(E)** Atacicept (200 µg) mixed with Flag-BAFF (10 µg). **(F)** APRIL/atacicept complex from fractions 10 and 11 of panel **(D)** were concentrated and analyzed again by size exclusion chromatography. The peak labelled “BSA” corresponds to bovine serum albumin of the elution buffer that had been co-concentrated with the complex. **(G)** Same as panel F, but for the BAFF/atacicept complex. Experiment was performed once in this format (twice for atacicept alone, atacicept plus BAFF and atacicept plus APRIL).

To estimate the stability of the BAFF-atacicept complex *in vivo*, atacicept alone or a mix of human Flag-BAFF plus atacicept were administered intravenously to mice. Mice were bled after 15 min, two days, or one to ten weeks after the injection. Interestingly, BAFF/atacicept complex could still be detected one month after the injection, while free atacicept was already below detection levels two days post-injection (**Supplementary Figures 2B, C**). The persistence of Flag-BAFF, Flag-APRIL or Flag-APRIL co-injected with atacicept in WT mice was also less than two days (**Supplementary Figures 2D–G**). To compare the persistence of pure BAFF/atacicept or APRIL/atacicept complexes in mice in the absence of free atacicept, these complexes were isolated by size-exclusion chromatography. Atacicept alone eluted in fractions 13 and 14, trimeric BAFF and APRIL alone eluted in fractions 14 and 15, and atacicept-BAFF and atacicept-APRIL complexes eluted in fractions 10 and 11 (**Figures 8C**, **9A–E**). When complexes were passed again on the size exclusion column, they were still present and stable as they contained all of detectable BAFF, APRIL and atacicept (**Figures 9F, G**). A mix of atacicept/APRIL and atacicept/BAFF complexes (1:1) was co-administered in mice for a direct comparison of their persistence in the circulation. APRIL was abundant after 15 min, still detectable at 4 h and disappeared thereafter while the co-administered BAFF/atacicept complex was readily detected at all time points, until the end of the experiment after one week (**Figure 8D**). In summary, co-injection data point to a difference in the half-life of ligand-bound atacicept in mice, with atacicept/BAFF complexes having a longer half-life than atacicept alone or atacicept/APRIL complexes, corroborating findings in humans (**Figures 2**, **5**).

## Discussion

A population PK model was developed using ‘total’ atacicept concentration data from 3 clinical trials as a framework to investigate the relationship between atacicept exposure and corresponding pharmacodynamic, efficacy and safety data. Kinetics of ‘free’ atacicept calculated with this population PK model aligned in relatively good agreement with values of ‘active’ atacicept measured in a reporter cell assay developed to circumvent the absence of a validated bioanalytical assay for unbound atacicept. This suggests that active atacicept can act as surrogate for free atacicept, and that the population PK model is sufficiently accurate to predict free respectively active atacicept levels. When total atacicept and active atacicept time concentration curves were compared in nine healthy volunteers after a single atacicept dose, the profiles revealed that active atacicept predominated up to 72 h post-dose, became half of total at around 96 h post-dose and remained meaningfully high for at least a week, i.e. with the ability to inhibit BAFF concentrations that would be at least 10 to 20 times higher than those prior to treatment initiation. This kinetics after a single dose of atacicept matched corresponding levels of unbound and complexed atacicept estimated by western blotting. It should be noted that drug-mediated pharmacodynamic (PD) changes depend on the amount of the active drug over time, and on the turnover rate of specific PD parameters such as immunoglobulins. Accordingly, in a Phase I study in healthy volunteers (EMR700461-022), a single atacicept dose reduced IgM and IgA whose half-lives are about 5 and 6 days, but not total IgG whose overall half-life is about 21 days (21). A notable decrease in subsets of B cells and in IgG levels, including disease-associated anti-dsDNA autoantibodies, required repeated atacicept treatment for 16 to 20 weeks. These parameters then remained reduced until cessation of treatment at week 52, after which time they gradually returned towards baseline levels (13).

In one SLE patient treated for 52 weeks, no active atacicept was detected at week 52, total atacicept was low, and atacicept/BAFF complexes detected by Western blot were like those at 12 weeks post-treatment. This patient was tested at week 24 for anti-drug antibodies and found to be negative. It is not known whether this outlier result reflects the true status of this patient at week 52 or a sample misidentification.

PK measures of total atacicept have long revealed a slow terminal elimination phase of atacicept (21, 36, 37) (and this study). Here we find that after a single dose of atacicept administered subcutaneously in healthy volunteers, serum levels of BAFF steadily increased up to 100-fold within 2 to 4 weeks, reaching several hundreds of ng/ml. Using theoretical molecular masses of 37 kDa for a monomer of atacicept with one N-glycosylation in the Fc portion, and of 17 kDa for a monomer of BAFF, the mass ratio of atacicept to BAFF is predicted to be 1.5 to 3 if one or two dimeric atacicept molecules bind to one BAFF 3-mer. Thus, atacicept/BAFF complexes alone can explain much of the total atacicept concentrations measured at late time points. In addition, a proportion of atacicept in serum is bound to a ligand that contains APRIL. Our results are consistent with this ligand being a BAFF-APRIL heteromer rather than a homo 3-mer of APRIL. It is presently unclear why APRIL, which can be readily detected in serum with the ELISA used in this study (32) does not increase after atacicept administration. Depletion of BAFF by atacicept may specifically stimulate BAFF synthesis so that newly produced APRIL would be preferentially incorporated into heteromers rather than into homo 3-mers. Alternatively, the half-life of APRIL in the circulation may not be markedly modified in the presence of atacicept. Indeed, APRIL binds to proteoglycans by surfaces distinct from those that mediate receptor binding (38), and APRIL co-localizes with proteoglycans in human arteries (32). APRIL can bind to proteoglycans even when bound to atacicept (32, 35). However, reversible binding of APRIL/atacicept complexes to proteoglycans in the vasculature seems unlikely since competing for APRIL binding to proteoglycans with heparin in mice that previously received atacicept failed to increase circulating atacicept levels (35). In a mouse model of SLE, APRIL was found to interact with a soluble form of the proteoglycan syndecan-1 (39). If atacicept-bound APRIL in humans further binds soluble syndecan-1, it may escape detection in our experimental systems. However, because interactions of APRIL with proteoglycan are relatively weak, anti-APRIL antibodies are likely to displace proteoglycans, should they recognize an overlapping binding site on APRIL. Our results comparing the fate of an atacicept/APRIL complex co-administered in mice with an atacicept/BAFF complex showed a rapid disappearance of APRIL versus a long persistence of BAFF, which is in line with observations in human serum samples. It is not known whether the SDS-stable, high molecular weight band of atacicept that is a “signature” of a BAFF/atacicept complex may be linked somehow to its long persistence, nor is it known whether similar complexes may form with endogenous TACI to modulate the function of TACI and/or the stability of BAFF *in vivo*.

Taken together, these data show that active atacicept monitored with the reporter cell-based assay can act as a surrogate for ‘free’ atacicept levels and mirrors the population PK model-derived free atacicept kinetics. Data also indicate complex relationships between atacicept and it targets with regard to distribution and/or stability issues: although it is expected that the half-life of a soluble cytokine increases after binding to a Fc-containing molecule, it was unexpected that this effect would be so different between APRIL and BAFF. It was also unpredicted that the long half-life of atacicept due to its Fc domain could be further increased by binding to BAFF. However, for the latter point, a direct comparison between free and BAFF-bound atacicept is complicated by the continuous conversion of the free to the bound form of atacicept in the presence of endogenous BAFF, which may artefactually reinforce the difference. It is noteworthy that the PK population model should not be significantly affected by differences between ligands as it was conceived to fit experimentally determined total atacicept levels. For this purpose, targets of atacicept were considered as a whole, without granularity for individual ligands or forms thereof.

In conclusion, the data support the utility of the population PK model and confirms the presence of an excess of active (free) atacicept at trough with the once-weekly dosing of atacicept used in clinical setting, which may also allow a longer dosing interval, *e.g.* every other week. However, this was not tested in clinical setting thus far. Finally, these data support future studies with atacicept and the characterization of its exposure-PD response relationship by combining data from the population PK model with PD markers (*e.g.* immunoglobulins, BAFF/APRIL, immune-cells, etc.) of relevance for the targeted indication(s).

## Data availability statement

The datasets presented in this study can be found online in the zenodo repository at doi: 10.5281/zenodo.7316264.

## Ethics statement

The studies involving human participants were reviewed and approved by the Office for Research Ethics Commitees Northern Ireland (study EMR700461-022) and by the relevant Institutional Review Boards (IRBS) or Independent Ethics Commitee (IECs) and by Health Authorities according to country-specific laws (study NCT00624338). Both EMR700461-022 (EudraCT ID: 2013-002703-34) and APRIL-SLE (NCT00624338) studies were conducted in accordance with their protocols, the International Conference on Harmonization (ICH) guideline for Good Clinical Practice (GCP) and applicable local regulations, as well as with the Declaration of Helsinki. The patients/participants provided their written informed consent to participate in this study. The animal study was reviewed and approved by the Office Vétérinaire Cantonal du Canton de Vaud (authorization 1370.8 to PS).

## Author contributions

ME, DW, ÖY and PS designed the study. ME, OP, LW and PS performed experiments and acquired data. DW, OD and ÖY contributed essential reagents. ME, ÖY and PS wrote the article. All authors contributed to the article and approved the submitted version.

## Funding

This study was supported by grants of the Swiss National Science Foundation (310030_156961, 31003A_176256 and 310030_205196 to PS) and by Merck Healthcare KGaA, Darmstadt, Germany (CrossRef Funder ID: 10.10339/100009945). Medical writing support was funded by Merck Healthcare KGaA, Darmstadt, Germany in accordance with Good Publication Practice (GPP3) guidelines (http://www.ismpp.org/gpp3). The funder was not involved in the study design, collection, analysis, interpretation of data, the writing of this article or the decision to submit it for publication.

## Acknowledgments

The authors thank the patients and their families, investigators, co-investigators, and the study teams at the participating clinical centers and at Merck Healthcare KGaA, Darmstadt, Germany, Merck Institute for Pharmacometrics, Lausanne, Switzerland, an affiliate of Merck KGaA, and EMD Serono Research & Development Institute, Inc., Billerica, MA, USA, an affiliate of Merck KGaA. In addition, the authors thank Bioscript Group Ltd, Macclesfield, UK for providing medical writing support in initial stages of this manuscript.

## Conflict of interest

PS was supported by a research grant from Merck Healthcare KGaA, Darmstadt, Germany. OP is employee of Merck Institute for Pharmacometrics, Lausanne, Switzerland, an affiliate of Merck KGaA. ÖY and DW were employee of Merck Healthcare KGaA, Darmstadt, Germany at the time of the study. OD was employed by Adipogen Life Sciences.

The remaining authors declare that the research was conducted in the absence of any commercial or financial relationships that could be construed as a potential conflict of interest.

## Publisher’s note

All claims expressed in this article are solely those of the authors and do not necessarily represent those of their affiliated organizations, or those of the publisher, the editors and the reviewers. Any product that may be evaluated in this article, or claim that may be made by its manufacturer, is not guaranteed or endorsed by the publisher.

## Abbreviations

APRIL, a proliferation-inducing ligand; BAFF, B cell activating factor of the TNF family; TACI, transmembrane activator and calcium-modulator and cyclophilin ligand (CAML)-interactor; SLE, systemic lupus erythematosus; PK, pharmacokinetic; PD, pharmacodynamic.
