# Supplementary material for: Kinetics of free and ligand-bound atacicept in human serum
Source: Front Immunol. 2022 Dec 2;13:1035556. doi: 10.3389/fimmu.2022.1035556 (PMC9756848; doi:10.3389/fimmu.2022.1035556)
Supplement: Supplementary file 1 [file DataSheet_1.pdf]

Supplementary Material

Supplementary Figures for Eslami et al.

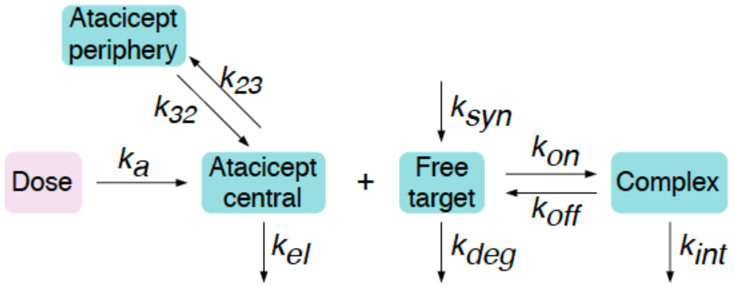

$$C = \frac{1}{2}\left[(C_{tot} - R_{tot} - k_{SS}) + \sqrt{(C_{tot} - R_{tot} - k_{SS})^2 + 4 \cdot k_{SS} \cdot C_{tot}}\right]$$

$$k_{SS} = \frac{k_{off}}{k_{on}} + \frac{k_{int}}{k_{on}}$$

$$\frac{dA_1}{dt} = -k_a \cdot A_1$$

$$\frac{dC_{tot}}{dt} = k_a \cdot \frac{A_1}{V_c} - (k_{el} + k_{23}) \cdot C - \frac{k_{int} \cdot C \cdot R_{tot}}{k_{SS} + C} + k_{32} \cdot \frac{A_3}{V_c}$$

$$\frac{dA_3}{dt} = k_{23} \cdot C \cdot V_c - k_{32} \cdot A_3$$

$$\frac{dR_{tot}}{dt} = k_{syn} - k_{deg} \cdot R_{tot} - (k_{int} - k_{deg}) \cdot \frac{C \cdot R_{tot}}{k_{SS} + C}$$

SUPPLEMENTARY FIGURE 1 | Schematic representation and differential equations of the two-compartment quasi steady-state target mediated drug disposition. C = free atacicept concentration, C_{tot} = total atacicept concentration, R_{tot} = total target concentration, k_{SS} = steady-state constant, k_{off} = dissociation rate constant, k_{on} = binding rate constant, k_{int} = drug-target complex elimination rate constant, k_a = absorption rate constant, A_n = amount in the nth compartment, k_{el} = elimination rate constant, k_{23} = transfer rate from central to peripheral compartment, k_{32} = transfer rate from peripheral to central compartment, V_c = volume of the central compartment, k_{syn} = target production constant, k_{deg} = target elimination constant.

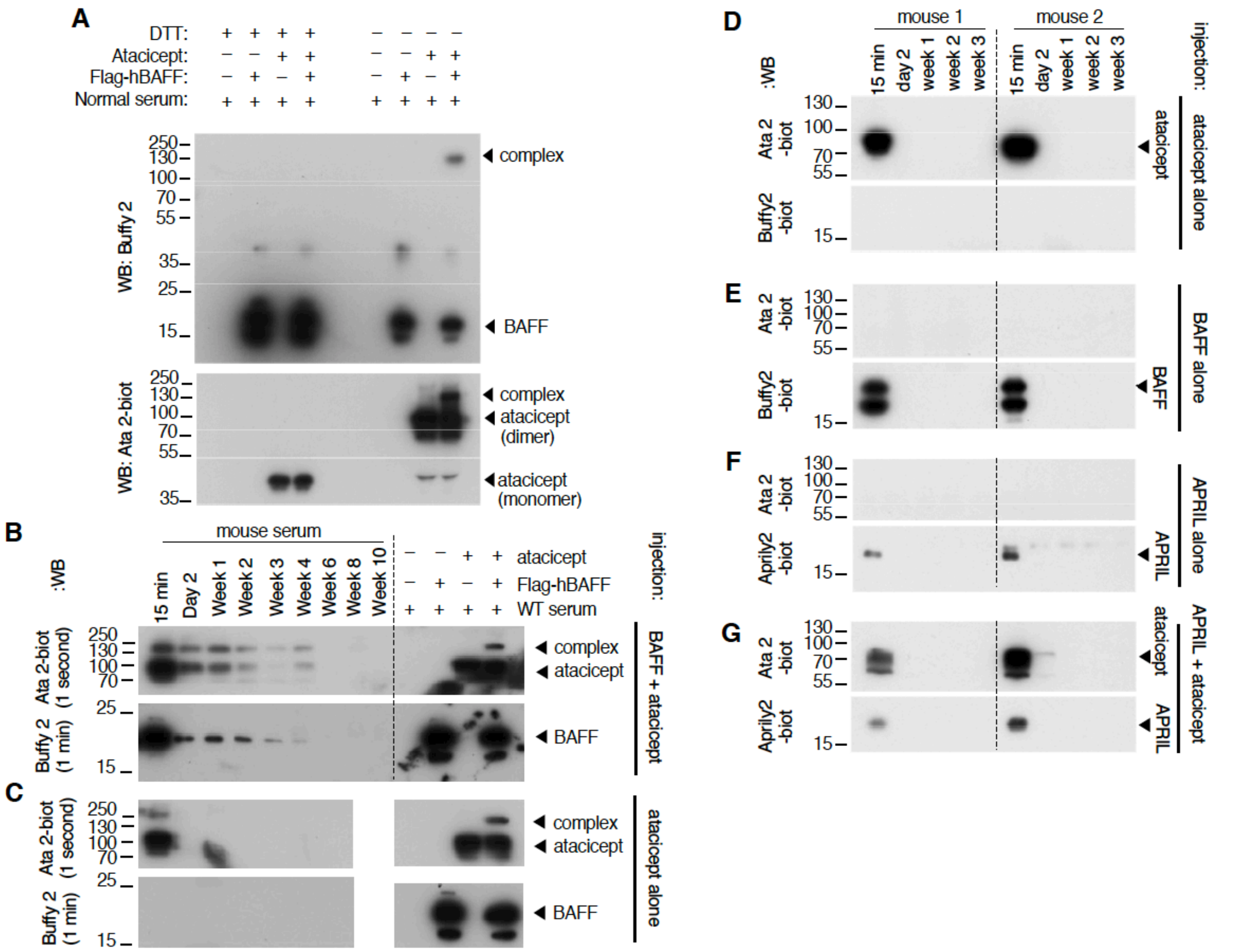

SUPPLEMENTARY FIGURE 2 | Stability of atacicept, BAFF, APRIL and complexes thereof in the mouse circulation. **(A)** Flag-BAFF (50 ng) co-incubated with atacicept (100 ng) was analyzed by SDS-PAGE under reducing or non-reducing conditions followed by Western blot with anti-BAFF mAb Buffy-2 or biotinylated anti-TACI mAb Ata 2. Experiment performed twice. **(B)** A mix of atacicept (200 µg) and Flag-BAFF (50 µg) co-administered intravenously to adult mice was subsequently detected by Western blot anti-TACI and anti-BAFF in serum samples prepared at the indicated time points. Results of one out of two experiments with similar results are shown. (**C)** Same as panel B, but with administration of atacicept (200 µg) alone. Result of one of two experiments is shown. **(D)** Same as panel C for two additional mice treated with atacicept (200 µg) alone. **(E)** Same as panel D, for mice treated with Flag-BAFF (50 µg) alone. **(F)** Same as panel D, for mice treated with Flag-APRIL (50 µg) alone. APRIL was detected by Western blot anti-APRIL. Experiments of panels D, E, and F were performed once. **(G)** Same as panel E, for mice treated with atacicept (200 µg) mixed with Flag-APRIL (50 µg). Experiment performed twice.
